# Unbiased RNA Shotgun Metagenomics in Social and Solitary Wild Bees Detects Associations with Eukaryote Parasites and New Viruses

**DOI:** 10.1371/journal.pone.0168456

**Published:** 2016-12-22

**Authors:** Karel Schoonvaere, Lina De Smet, Guy Smagghe, Andy Vierstraete, Bart P. Braeckman, Dirk C. de Graaf

**Affiliations:** 1 Laboratory of Molecular Entomology and Bee Pathology, Department of Biochemistry and Microbiology, Faculty of Sciences, Ghent University, Ghent, Belgium; 2 Laboratory of Agrozoology, Department of Crop Protection, Faculty of Bioscience Engineering, Ghent University, Ghent, Belgium; 3 Laboratory of Ageing Physiology and Molecular Evolution, Department of Biology, Ghent University, Ghent, Belgium; Louisiana State University, UNITED STATES

## Abstract

The diversity of eukaryote organisms and viruses associated with wild bees remains poorly characterized in contrast to the well-documented pathosphere of the western honey bee, *Apis mellifera*. Using a deliberate RNA shotgun metagenomic sequencing strategy in combination with a dedicated bioinformatics workflow, we identified the (micro-)organisms and viruses associated with two bumble bee hosts, *Bombus terrestris* and *Bombus pascuorum*, and two solitary bee hosts, *Osmia cornuta* and *Andrena vaga*. Ion Torrent semiconductor sequencing generated approximately 3.8 million high quality reads. The most significant eukaryote associations were two protozoan, *Apicystis bombi* and *Crithidia bombi*, and one nematode parasite *Sphaerularia bombi* in bumble bees. The trypanosome protozoan *C*. *bombi* was also found in the solitary bee *O*. *cornuta*. Next to the identification of three honey bee viruses Black queen cell virus, Sacbrood virus and Varroa destructor virus-1 and four plant viruses, we describe two novel RNA viruses Scaldis River bee virus (SRBV) and Ganda bee virus (GABV) based on their partial genomic sequences. The novel viruses belong to the class of negative-sense RNA viruses, SRBV is related to the order Mononegavirales whereas GABV is related to the family *Bunyaviridae*. The potential biological role of both viruses in bees is discussed in the context of recent advances in the field of arthropod viruses. Further, fragmentary sequence evidence for other undescribed viruses is presented, among which a nudivirus in *O*. *cornuta* and an unclassified virus related to Chronic bee paralysis virus in *B*. *terrestris*. Our findings extend the current knowledge of wild bee parasites in general and addsto the growing evidence of unexplored arthropod viruses in valuable insects.

## Introduction

Bees are valuable insects because of their dedicated role in the pollination of flowering plants [1, 2]. The domesticated western honey bee, *Apis mellifera*, has a central role in commercial pollination and is traditionally employed by farmers to enhance crop yields [3]. More recently, however, also bumble bees and even solitary bees—which are collectively referred to as wild bees—find their way into commercial environments as alternative or supplemental crop pollinators. For example, the Megachilid bees *Osmia bicornis* and *Osmia cornuta* are reared and utilized for orchard pollination in Western-European countries [4]. Solitary bees are a species-rich group of valuable wild insects in terms of pollination ecosystem services [1] and the conservation of plant diversity [5].

The recent trend in pollinator declines prompted researchers to investigate the contributory stressors to bee health [5–7]. Hence, the adverse interaction of bees with parasites and viruses is regarded as a major stressor [8]. Bee eukaryote parasites in general are taxonomically diverse including trypanosomes, apicomplexans, microsporidians, ascomycete fungi and also higher eukaryotes including nematodes, mites, flies and wasps [9]. Host-parasite interactions are well studied for most of the parasites in *A*. *mellifera* [10] and for two bumble bee parasites, *Crithidia bombi* [11] and *Nosema bombi* [12]. Some bee parasites have already been discovered in the past century [13, 14] or even before [15], but now have faded into the background. Very recently, a *Tubulinosema* parasite was discovered infecting bumble bees in Argentina [16].

Next to eukaryote parasites, also pathogenic viruses are known to affect social bees. Honey bees can be overwhelmed by 24 virus species including positive-sense (+) single-stranded (ss) RNA viruses of the orders *Picornavirales* and *Tymovirales*, and two double stranded (ds) DNA viruses of the families *Baculoviridae* and *Iridoviridae* [17]. Multiple honey bee RNA viruses are prevalent in sympatric honey bee and bumble bee populations [18] and some are infectious towards *Bombus* spp. [19–21]. Together, these studies indicate that many bee viruses have a broad host range. No specific virus has as yet been described from solitary bees, although multiple occurrences of honey bee viruses in solitary bees have been reported [22, 23]. Contrary to their typical implication in bee pathology, viruses may also adopt a symbiotic or mutualistic lifestyle with an insect host [24]. For example, polydnaviruses are essential to parasitoid wasps as they ensure egg survival by shutting down the immune system of an infected victim [25]. In contrast to the restricted taxonomic diversity of the currently known bee viruses, arthropod viruses are found in many other viral taxa. The rapid succession of viral metagenomics studies during the last 10 years has blurred the taxonomic delineation of viral families that infect arthropod hosts. A growing number of studies discovering new arthropod viruses challenge the idea that we have about viral diversity and evolution [26, 27]. In this regard, the exponential increase of viral sequences in public repositories has a knock-on effect on the identification of novel viruses both in existing [28] and new high-throughput sequencing datasets.

The current central workflow in metagenomics includes parallel sequencing of small pieces of DNA or cDNA up to a few hundred nucleotides followed by bioinformatics analysis. Two main strategies exist that differ in either being targeted or untargeted. In the targeted strategy, or amplicon-based metagenomics sequencing, a defined sequence that is shared among all organisms of interest such as the 18S ribosomal DNA is amplified and sequenced. A limitation of amplicon sequencing is that viruses or unrelated eukaryote organisms are excluded from detection [29]. The untargeted strategy or shotgun metagenomics sequencing (SMS), can circumvent this limitation by random amplification of genetic material prior to sequencing. The aim of this study was to explore the diversity of eukaryote microorganisms and viruses that are associated with wild bees. Since bees are known to harbor RNA viruses and far-evolved microsporidians, we opted for RNA SMS. Moreover, this strategy allows the simultaneous detection of the host and parasite transcriptomes.

## Materials and Methods

### Specimen collection

Wild bees were collected during spring 2015 in Ghent (Belgium, 51°01'30.5"N 3°42'45.9"E) within 500 meter distance of the apiary of Honeybee Valley at Ghent University. Sampling authorization and ethics approval were not required as no endangered or protected species were collected. We only sampled on days with favorable weather conditions to ensure high bee activity (sunny, less than 50% cloud coverage, more than 20°C and wind velocity less than 5 bft). Between five and ten individuals of the four wild bee species *Bombus terrestris*, *Bombus pascuorum*, *Osmia cornuta* and *Andrena vaga* were caught using an insect aerial net, immediately transferred to a vial and euthanized by placing the vials on dry ice. All bees were of the female sex and in case of bumble bees we only sampled queens. The sample size of each bee species is limited by the number of bumble bee queens because they are relatively rare in nature. Moreover, *Bombus terrestris* is one of four cryptic species of the subgenus *Bombus sensu stricto* in Belgium which are hard to distinguish morphologically. All subject individuals appeared healthy at the time of sampling. Bumble bees were caught on the fly or while visiting flowering plants. Solitary bees were caught in the proximity of the nest entrance.

### Species determination

Species determination was confirmed by amplification of the cytochrome C oxidase subunit I (COI) DNA barcode region. Genomic DNA (gDNA) was extracted from one middle leg based on the procedure described in [30]. Primers used for PCR amplification were LCO1490 5’-TATCAACCAATCATAAAGATATTGG and HCO2198 5’-TAAACTTCAGGGTGACCAAAAAATCA [31]. The PCR reaction mixtures contained 2 μM of each primer, 1 mM MgSO_4_, 0.2 mM dNTPs, 10x Pfx amplification buffer, 1 U Platinum® Pfx DNA polymerase (Thermo Fisher Scientific, Cat. No. 11708–013) and 1 μl template gDNA. The PCR reaction conditions were 95°C for 5 min, 35 cycles of 94°C for 15 s, 50°C for 30 s, 68°C for 45 s, and a final elongation step at 68°C for 10 min. PCR products were submitted to GATC Biotech AG (Constance, Germany) for dideoxy DNA sequencing in both directions. The sequences were queried against the Barcode of Life Data (BOLD) Systems COI database for species identification [32].

### Sample preparation

Bee abdomens were microscopically examined and present ectoparasitic mites and pollen were removed. The bee thorax was dissected on a minus 80°C surface and only the head and abdomen tagmata were kept for further analysis. Tissue was homogenized in 1 ml QIAzol Lysis Reagent (QIAGEN) using a Precellys®24 instrument for 90 s at 5 kHz in the presence of zirconium and steel beads. Total RNA was extracted using the QIAGEN RNeasy Lipid Tissue Mini kit. Residual DNA was removed by an on-column DNase I treatment using the QIAGEN RNase-free DNase set. Subsequently, total RNA of 5 individuals of the same species was pooled (10 μg of each individual) and 50 μg pooled total RNA was subjected to polyA+ mRNA enrichment using the QIAGEN Oligotex mRNA Mini kit. All protocol steps were performed according to manufacturer’s instructions. Prior to sequencing, the quality and concentration of total RNA and polyA+ mRNA samples was determined by capillary electrophoresis on a Bioanalyzer instrument using the RNA 6000 Pico kit (Agilent).

### Reverse transcription and PCR assays

One μl of pooled total RNA was reverse transcribed using RevertAid First Strand cDNA Synthesis Kit (Thermo Fisher Scientific) according to manufacturer’s instructions. The cDNA was subjected to various in-house PCR assays for viral and eukaryote bee pathogens as detailed in previous work [23]. Targets were Deformed wing virus/Varroa destructor virus-1 (DWV/VDV-1), Black queen cell virus (BQCV), Lake Sinai virus (LSV), Apis mellifera filamentous virus (AmFV), Varroa destructor macula-like virus (VdMLV), Sacbrood virus (SBV), *Apicystis bombi*, *Crithidia bombi*, *Nosema* spp. (including *Nosema apis*, *Nosema bombi* and *Nosema ceranae*), and *Ascosphaera apis*. For primer sequences, see S7 File. PCR reactions for the detection of new viruses contained 10x PCR buffer, 1 mM MgCl_2_, 200 μM of each dNTP, 2 μM of each primer, 1 unit HotStartTaq polymerase (QIAGEN) and 10 to 100 ng of cDNA template. Thermal cycling conditions were 95°C for 5 min, 35 cycles of 94°C for 30 s, 55°C for 30 s, 72°C for 60 s, and a final elongation step at 72°C for 10 min.

### Library preparation and Ion torrent semiconductor sequencing

All reagents and kits for library preparation and sequencing were obtained from Life Technologies. One hundred ng polyA+ mRNA was subjected to stranded cDNA library preparation according to the barcoded whole transcriptome library protocol using the Ion Total RNA-Seq Kit v2 and Ion Xpress™ RNA-Seq Barcode 1–16 Kit. Briefly, 100 ng polyA+ mRNA was fragmented by 1/10 of the recommended volume RNase III enzyme for 30 s, fragments were ligated to adaptor sequences and cDNA was synthesized. The resulting four cDNA construct libraries were purified, separately barcoded and amplified by eight PCR cycles. Amplified cDNA construct libraries were subsequently selected for a 400 bp insert size, pooled and prepared for clonal amplification on Ion Sphere™ Particles by emulsion PCR with the 400 bp template kit. Finally, clonally amplified cDNA constructs were loaded onto one Ion 318™ Chip v2 for semiconductor sequencing on the Ion Torrent Personal Genome Machine (PGM) System with the 400 bp sequencing kit.

### Sequence assembly, annotation and taxonomic profiling

Raw sequencing reads were uploaded to Torrent_Suite v4.4.3 portal for adapter trimming and demultiplexing of barcoded reads. The quality of the raw sequence data was inspected with the FastQC tool [33]. Additional quality metrics were applied using CLC Genomic Workbench 8.5 software’s trim sequences tool with default parameters and minimum length 50. High quality reads were de novo assembled into contigs using the CLC de novo assembler and contigs longer than 200 bps were retained. Reads were mapped back to contigs to improve the assembly result. The superset of contigs and unassembled reads equal or longer than 100 nt concluded the set of tentatively unique genes (TUGs). TUGs were queried against the latest versions of the nucleotide and non-redundant protein sequence databases using blastn (blast+ version 2.2.31) and diamond blastx [34], respectively. The parameters for blastn were adjusted to allow for somewhat similar sequence hits: word size 11, match 2, mismatch -3, gap open cost 5 and extend cost 2, expect threshold 1e-6. The 20 top hits were retained. To speed up analysis time, queries were split-processed using GNU Parallel [35] and eight 24-core processors on the Stevin Supercomputer Infrastructure (Ghent). Diamond blastx analysis was run in sensitive mode and E-value threshold of 1e-05. The annotated TUGs were taxonomically binned with the latest MEGAN software version [36], which uses a lowest common ancestor (LCA) algorithm to assign reads to the NCBI taxonomy hierarchy. The LCA algorithm parameters were adjusted to Min Score 200 (nucleotide) or 50 (protein), Top Percent 10 and Min complexity 0.3. For each metagenome, a taxonomic profile was computed using the Projection Method at the family rank and Min Support Percent 1e-03. Profiles as thus generated were compared and visualized in MEGAN6.

### Sanger validation of viral genomic sequences

Viral reads and contigs were used to design primers to confirm the presence of the virus and to fill in the missing gaps in the genomic sequence (S1–S4 Files). All primer sequences used for validation and RT-PCR of the new viruses are summarized in S5 File. The Expand Long Template PCR System (Roche) was used to amplify fragments up to 4 kbp of viral cDNA. The reaction mix contained 10x buffer 2, 350 μM of each dNTP, 3.75 U polymerase, 300 nM of each primer, up to 500 ng template cDNA and adjusted to 50 μl with distilled DNase/RNase-free water (Life Technologies). Thermal cycling conditions were 2 min denaturation at 95°C, 10 initial amplification cycles of 10 s at 94°C, 30 s at 55°C and 4 min at 68°C followed by 25 additional cycles incrementing elongation time each cycle by 30 s. The reaction was finalized by 7 min elongation at 68°C. Long template products were sent to GATC Biotech (Constance, Germany) for Sanger sequencing of overlapping fragments up to 850 bp in both orientations. Templates that failed sequencing were ligated into pCR®4-TOPO® Vector (Life Technologies) after a 10 min 72°C incubation step with Taq polymerase (Qiagen) to ensure the presence of 3’-A overhangs. Further cloning steps were carried out with One Shot® TOP10 chemically competent bacteria cells (Life Technologies) according to manufacturer’s instructions. Plasmids were isolated using the GeneJET Plasmid Miniprep Kit (Thermo Scientific) starting from a 5 ml culture grown overnight. Purified plasmids were sent to GATC Biotech in the specified concentration range and were sequenced using M13 or custom primers. The sanger fasta sequences were trimmed and assembled in CLC Genomics Workbench.

### Phylogenetic analysis

Open reading frames (ORFs) were predicted using the freely available software FgenesV0 or translated using the ExPASy translate tool. Reference amino acid sequences of related viruses were obtained from GenBank. Multiple sequences alignments of RNA-dependent RNA polymerase (RdRp) proteins were constructed using the MAFFT version 7 and the E-INS-i algorithm [37]. The alignment quality was evaluated using the TCS strategy [38] as part of the t-coffee software. Columns with a score lower than 5 were removed. Next, the alignment was manually inspected for correct aligning of conserved residues and trimmed if necessary. For SRBV and GABV, respectively, 545 and 512 amino acid (aa) positions were present in the final alignment. ProtTest3 v3.4.2 [39] was used to select the best-fit model of protein evolution among 120 candidate models starting from a fixed BIONJ JTT tree topology. Both maximum likelihood (ML) and Bayesian inference (BI) analyses were carried out to reconstruct evolutionary trees under the LG substitution model with optimized invariable sites (+I), a discrete Gamma distribution (+G) of rates over sites with 4 rate categories and empirical equilibrium aa frequencies (+F). For the ML analysis, the consensus tree was inferred from 1 000 bootstrap replicates using PhyML v3.1 [40] embedded in SeaView [41]. Using the MrBayes software v3.2.6 [42], two parallel Metropolis-coupled Markov chain Monte Carlo runs were conducted for 1M generations (split frequencies < 0,01) and the tree was summarized after discarding the first 100K runs (10% burn-in).

### GenBank depositions

The partial genomic sequence of SRBV was deposited in GenBank under accession number KY053857. The full L segment of GABV and the partial sequences of the M and S segments were deposited under accession numbers KY053854-6, respectively.

## Results

### Data quality and Ion torrent semiconductor sequencing

In order to verify the quality of the samples and the success of polyA+ mRNA enrichment, we analyzed the RNA profile of total and polyA+ mRNA enriched samples by capillary electrophoresis. The profiles of polyA+ mRNA samples showed a strong reduction of rRNA peak sizes and a general decrease in the rRNA/mRNA ratio compared to the total RNA samples (S6 File). In both *Bombus* species, the profiles showed an increased signal of the LSU peak compared to solitary bees which suggested the abundant presence of non-insect eukaryote organisms. Subsequent Ion Torrent semiconductor sequencing of the multiplexed cDNA libraries generated 4 412 402 raw reads and a total of 3 876 076 high quality reads (average read length = 247 bps) were retained after applying stringent quality metrics. Statistics for semiconductor sequencing and de novo assembly are summarized in Table 1. Although normalized RNA amounts were used for cDNA library construction, the number of high quality reads differed substantially between datasets (lowest 706 426 for *B*. *pascuorum* and highest 1 387 148 for *B*. *terrestris*). Between 56% and 80% of the TUGs were successfully annotated. An unquantifiable fraction of the reads were chimeric constructs that likely originated during adapter ligation (through insert fusion) or PCR. Chimera construct formation has been reported in another RNA-seq study [43]. We further discuss this error in S6 File. Further, we observed many occurrences of homopolymer-associated indel errors and the majority of errors were observed for the nucleotides A and T. Homopolymer indel errors complicated downstream blastx analysis through the introduction of frameshifts. However, errors did not impede the inference of biological function from the data.

**Table 1:** Summary statistics of Ion Torrent semiconductor sequencing and de novo assembly.

Host	*B*. *terrestris*	*B*. *pascuorum*	*O*. *cornuta*	*A*. *vaga*
Raw reads	1 717 200	773 550	1 082 096	839 556
High-quality reads	1 387 148	706 426	998 535	783 967
Contigs	31 462	24 459	21 441	17 785
Unassembled reads	178 512	127 399	103 051	81 651
Total TUGs^A^	209 974 (64%)	151 858 (80%)	103 051 (73%)	99 436 (56%)

^A^TUG = Tentative Unique Gene; percentage successfully annotated is indicated between brackets

### Taxonomic profile of organisms associated with wild bees

**Fig 1:**
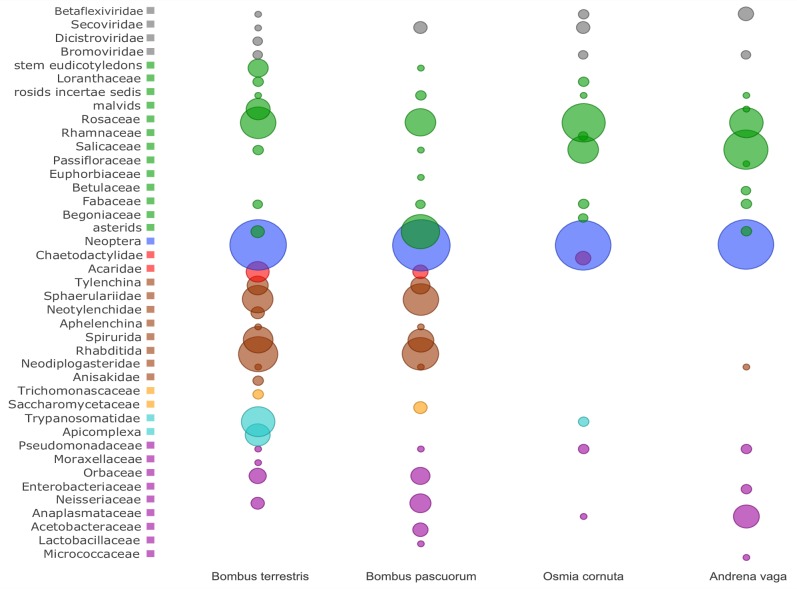
Schematic representation of the different communities in *Bombus terrestris*, *Bombus pascuorum*, *Osmia cornuta* and *Andrena vaga* metagenomes. Circles represent the relative number of contigs and reads assigned to a particular taxon (left) by MEGAN6 analysis. Taxa clustering in the same Phylum or community were given the same colour: Viruses (grey), Plants (green), Host (blue), Mites (red), Nematodes (brown), Fungi (orange), Protists (turquoise) and Prokaryotes (magenta).

MEGAN analysis of blastn annotated TUGs accommodated eight composite communities in wild bees (Fig 1). Most of the TUGs clustered in Neoptera or below and no other species than the host could be ascertained from this community. In total, 815 TUGs were assigned to Bacteria, 1 379 to Protozoa, 10 104 to Nematoda, 197 to Acari (mites), 89 to Fungi, 23 872 to Plants and 110 to Viruses. The number of TUGs assigned to Viruses was higher (178 TUGs) and more accurate for protein annotations than for nucleotide annotations. The other communities did not benefit from the protein annotations as respective numbers of assigned TUGs decreased dramatically.

The protozoan community was composed of two distinct lineages in Alveolata and Euglenozoa. In the Alveolata lineage, 113 *B*. *terrestris* TUGs clustered in Apicomplexa. Close examination of the partially reconstructed SSU rRNA TUGs revealed 99% identity to *Apicystis bombi*, a known parasite of bumble bees and honey bees. In Euglenozoa, 870 TUGs of *B*. *terrestris* and 15 TUGs of *O*. *cornuta* clustered in *Trypanosomatidae* (Euglenozoa). The partially reconstructed SSU rRNA in both metagenomes revealed 100% identity with *Crithidia bombi*, which is a known parasite of bumble bees. Other sequences for heat-shock proteins, ribosomal proteins and core carbohydrate metabolism generally clustered within the *Leishmania-Crithidia* lineage, confirming the taxonomic position of this protozoan. RT-PCR analysis confirmed the presence of *A*. *bombi* in *B*. *terrestris* but not the presence of *Crithidia* in *B*. *terrestris* and *O*. *cornuta* (S3 File).

The fungal community predominantly comprised yeasts of the order of Saccharomycetales or budding yeasts. Ten TUGs of *B*. *terrestris* clustered in the family *Trichomonascaceae* with a mean identity of 92% to the LSU rRNA of *Wickerhamiella* sp. One single TUG of 109 nt showed 99% identity to *Candida bombiphila* LSU rRNA. Among the many TUGs that clustered in the *Saccharomycetaceae* family in *B*. *pascuorum*, none could be assigned to a specific species. No microsporidian associates were detected by RNA SMS. However, a broad-spectrum *Nosema* PCR assay was positive in *B*. *terrestris*, *O*. *cornuta* and *A*. *vaga* metagenomes which suggests that our strategy lacked the analytical depth to detect low abundant associations. Sanger sequencing of the PCR products showed 99% identity to *Nosema thomsoni* (gb|KC596023.1).

Two nematode species and several mite species were detected by RNA SMS. Between three and five percent of TUGs in both *Bombus* species clustered diversely in the nematode phylum. Close examination of the partially reconstructed SSU and LSU rRNA TUGs showed 99% identity to *Sphaerularia bombi*, a known parasite of bumble bee queens. The SSU rRNA sequences of the *B*. *terrestris* and the *B*. *pascuorum* associated *Sphaerularia* were nearly identical, however, both were higher to an isolate from Japan (99%, gb|AB250212.1) than to an isolate from the United Kingdom (96%, gb|AB250213.1). After revision of available sequence data, the UK *S*. *bombi* isolate showed 99% nucleotide identity to the Japanese hornet parasite *Sphaerularia vespae* (gb|AB300595.1). Non-ribosomal TUGs abundantly clustered in diverse orders in the nematode phylum because no reference transcriptome of a close relative to *S*. *bombi* was available at the time of study. Further, two TUGs from *A*. *vaga* clustered in *Neodiplogasteridae* with near perfect identity to the LSU rRNA sequence from a *Koerneria* sp. of unknown origin (gb|EU195999). Despite of our effort to remove ectoparasitic mites prior to sample preparation, one hundred thirteen TUGs from *B*. *terrestris*, 24 from *B*. *pascuorum* and 24 from *O*. *cornuta* clustered in Acari. We were unable to discriminate either one or multiple mite species in *B*. *terrestris*. All sequences clustered in the family *Acaridae* and TUGs for partially reconstructed LSU rRNA showed 99 percent identity to *Tyrophagus longior* and *Acarus* sp. The mite species associated with *O*. *cornuta* was highly related to *Chaetodactylus krombeini* (*Chaetodactylidae*), a pest mite of the orchard pollinator *Osmia cornifrons* [44].

The polyA+ mRNA enrichment removed near all bacterial mRNA transcripts. Nevertheless, the most abundant bacterial species were identified based on ribosomal RNA sequences. The bacterial community consisted of the characteristic gut flora of *Bombus* [45, 46]. The genera *Snodgrasella* (*Neisseriaceae*) and *Gilliamella* (*Orbaceae*) were detected in both *Bombus* metagenomes. The genera *Lactobacillus* (*Lactobacillaceae*), *Acetobacter* and *Gluconobacter* (*Acetobacteraceae*) were associated with *B*. *pascuorum* and *Acinetobacter* (*Moraxellaceae*) with *B*. *terrestris*. None of these bacterial genera in *Bombus* were found in solitary bees. The obligate endosymbiont *Wolbachia* (*Anaplasmataceae*) was found in *O*. *cornuta* and *A*. *vaga*. The genera *Arthrobacter* (*Micrococcaceae*) and *Escherichia* (*Enterobacteriaceae*) were only found in the ground nesting *A*. *vaga*.

The plant community correctly represented the flower preferences of the wild bees. In the studied area *B*. *pascuorum* (but also *B*. *terrestris*) foraged most of the time on *Glechoma hederacea* (Astrids) whereas *B*. *terrestris* was frequently visiting *Chelidonium majus* (stem eudicotyledons). Many stone fruit trees were flowering at the time of sampling and it is apparent from the data that all bees were foraging on *Prunus* sp. but also other *Rosaceae* such as plants of the tribe *Maleae*. In contrast to bumble bees, solitary bees had many hits to the genus *Salix (Salicaceae*), especially *A*. *vaga*. Indeed, solitary bees are in strong demand of pollen for nest care and therefore prefer plants that supply vast amounts of pollen such as willows. Identification of plants to the species level was not possible except for *Chelidonium majus* in *Bombus* and *Malus domestica* var. ‘Golden Delicious’ in *O*. *cornuta*. Many of the pollen-derived transcripts had functions in pectin metabolism including pectinesterases, pectate lyases, polygalacturonases and pectin methylesterases or coded for pollen-specific proteins.

### Genomic characterization of two new RNA viruses in *Osmia cornuta*

In the *O*. *cornuta* dataset, 56 TUGs corresponding to 1 041 ion torrent reads (0.1%) showed similarity to viral polymerase proteins. Taxonomic analysis allocated the TUGs into negative-sense ssRNA virus taxa. Using the 56 TUGs as a starting point, primers were designed to fill in the missing gaps by RT-PCR and the resulting amplicons were re-sequenced by the Sanger method. Two new viruses could be ascertained and they were assigned tentative names Scaldis River bee virus (SRBV) and Ganda bee virus (GABV), both names referring to the location where the bees were collected. Phylogenetic analysis based on the RdRp proteins placed SRBV among recently discovered arthropod chuviruses of the order Mononegavirales (S1 Fig) whereas GABV was placed among the informal phasmaviruses in the family *Bunyaviridae* (S2 Fig).

**Fig 2:**
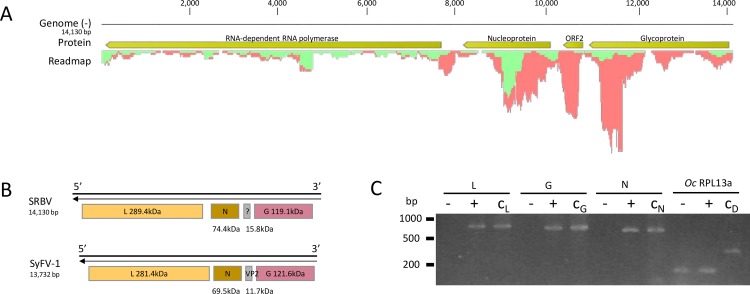
Organization and characteristics of the unsegmented Scaldis River bee virus (SRBV) genome. **-A)** High quality reads were mapped against the validated, near full-length genome sequence of SRBV using the CLC Genomic Workbench 8.5 mapping tool. Predicted ORFs that encode putative viral proteins were manually annotated (yellow). The readmap depicts the nucleotide coverage by viral genomic RNA (green) and complementary cRNA (red) reads. Note the relatively higher cRNA fraction towards the 3’ end of the genome.–**B)** Comparison of the genome organization of SRBV and its closest relative Shayang Fly Virus 1 (SyFV-1). RdRp (L), nucleoprotein (N), ORF2/VP2 (?) and glycoprotein (G) have similar positions and mass ranges.–**C)** RT-PCR amplification of 700–800 bp regions of the L, G, N genes and a housekeeping gene RPL13a in a SRBV-negative *Osmia cornuta* female (-) compared to a SRBV-positive female (+). Control samples C_L_, C_G_ and C_N_ were viral RT-PCR products generated during sanger validation. The RPL13a control C_D_ is a DNA extraction sample of a bee leg. RPL13a intron-spanning primers were specifically designed to discriminate gDNA (313 bp) from cDNA (119 bp).

We obtained a single segment of 14 130 nt representing an almost complete genome sequence of SRBV (Fig 2A). Bioinformatics analysis predicted the presence of at least four non-overlapping ORFs, three of which encode major viral proteins including the RdRp protein of 2 533 aa, a putative nucleoprotein of 659 aa and a putative glycoprotein of 1 042 aa. A fourth predicted ORF of 138 aa was situated in between the nucleoprotein and the glycoprotein ORFs, however, no biological function could be assigned based on protein homology. Blastp analysis of the full-length SRBV RdRp protein, nucleoprotein and glycoprotein queries showed only 38%, 25% and 20% identity respectively to the proteins of its closest relative Shayang Fly Virus 1 (SyFV-1). The genome organization of SRBV is similar to that of SyFV-1 (Fig 2B). Since strand orientation was preserved during NGS library preparation, a clear distinction could be made between viral genomic reads (vRNA) and complementary strand reads (cRNA). Fig 2A depicts a readmap illustrating the differential coverage of SRBV vRNA and cRNA reads. For the RdRp ORF, equal number of reads from both strands were detected, however, for the other ORFs, the relative number of cRNA reads was higher. These results indicate that the virus is actively replicating and that there was a relative higher transcript abundance of the 3’ end ORFs. None of the candidate L, G and N ORFs were shown to be part of the *O*. *cornuta* transcriptome (Fig 2C).

**Fig 3:**
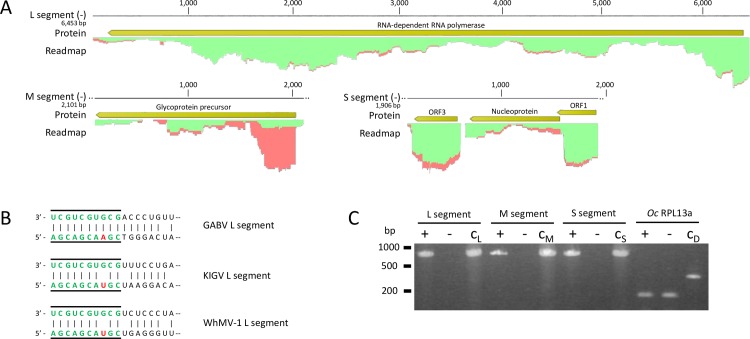
Organization and characteristics of the segmented Ganda bee birus (GABV) genome. –**A)** similar workflow as in Fig 2A; predicted ORFs are indicated in yellow. Coverage of viral genomic RNA (green) is higher than complementary cRNA (red) for the L and S segments, but vice versa for the 3’ end of the M segment.–**B)** The GABV 5’ and 3’ terminal regions are depicted and show strong complementarity which is typical for segmented bunyaviruses. The terminal regions of two other phasmaviruses are identical in the first 10 residues (green/line) except for pos. 7 (red). The terminal repeats are present on each segment and are involved in the regulation of genome replication in bunyaviruses. The terminal regions of the M and S segments were not found.–**C)** similar as in Fig 2C; samples are RNA extractions from the same GABV-negative (-) or GABV-positive (+) female bee. All three segments are absent in a GABV-negative individual and therefore not part of the *O*. *cornuta* transcriptome. KIGV = Kigliuak phantom virus; WhMV-1 = Wuhan Mosquito Virus 1; RPL13a = Ribosomal Protein L13a.

GABV is related to the family *Bunyaviridae* and like other bunyavirids, its genome is organized in three segments L, M and S. Since there was no direct evidence for the bunyavirid M and S segments, we performed tblastn searches against the *O*. *cornuta* dataset using M and S encoded proteins of related bunyaviruses as queries. Two contigs were identified as candidate bunyavirid M and S segments. The contigs matched hypothetical proteins from *Habropoda laboriosa*, *Dufourea novaeangliae* and *Bombus terrestris* after blastx analysis. The candidate L, M and S sequences were not part of the *O*. *cornuta* transcriptome as demonstrated by RT-PCR (Fig 3C). This result, however, does not imply that the three separate RNA molecules originate from a single virus. Following Sanger validation, the partial genomic segments of GABV were 6 453, 2 101 and 1 906 nt in size. The L and M segments each contained one ORF whereas three ORFs were predicted from the S segment (Fig 3A). Blastp analysis of the full-length GABV RdRp protein (2 087 aa) displayed 39 to 41% identity over the full query range to the RdRp protein of Wuchang Cockroach Virus 1, Seattle Prectang virus and Wuhan Mosquito Virus 1. The glycoprotein precursor (673 aa) and nucleoprotein (312 aa) queries respectively showed 26 to 32% and 32 to 35% to the same viruses. All three segments displayed 25% to 35% identity with Kigluiak phantom virus. Further, the GABV L segment was potentially complete since complementary regions at the sequence termini could be identified. These conserved nucleotide patterns are a characteristic feature of bunyavirid genome segments. Moreover, the terminal decanucleotide sequence of GABV (5’-UCGUCGUGCG) was identical to that of other phasmaviruses (Fig 3B). The complementary regions were not found on the M and S segments, indicating that these segments were only partially recovered. The read maps in Fig 3A demonstrated that both vRNA and cRNA reads were present, indicating that the virus was actively replicating.

### Detection of honey bee viruses, plant viruses and other viral sequences

A total of 4 468 reads (0,12%) assembled into 178 TUGs, which clustered into the viral community. Of the known viruses, we detected three honey bee viruses and four plant viruses. Honey bee-pathogenic RNA viruses were found in *B*. *terrestris* and *O*. *cornuta*. One TUG had 98 percent identity to SBV (*Iflaviridae*) and two TUGs had 95 and 100 percent identity to BQCV (*Dicistroviridae*) in *B*. *terrestris*. Two TUGs had 70 and 93 percent identity to VDV-1 (*Iflaviridae*) in *O*. *cornuta*. All reads were of the genomic orientation. RT-PCR analysis confirmed the presence of SBV and VDV-1, however, the presence of BQCV could not be confirmed by a PCR assay capable of detecting honey bee-associated BQCV (S7 File). Four (+) ssRNA plant viruses were detected and three of them were found in all bee species: Cherry leaf roll virus (*Secoviridae*, order Picornavirales), Prune dwarf virus (*Bromoviridae*) and Cherry A virus (*Betaflexiviridae*, order Tymovirales). The fourth plant virus, Arabis mosaic virus (*Secoviridae*) was only detected in *B*. *pascuorum*. Further, 22 TUGs deriving from all metagenomes except *A*. *vaga* showed varying but generally low degree of similarity to the genus *Fabavirus* (*Secoviridae*). Recurring top hits were Gentian mosaic virus, Lamium mild mosaic virus and Broad bean wilt virus 1. No reads of the negative-sense orientation were detected in the plant viruses.

TUGs and corresponding blastx hit statistics for SRBV, GABV and other unknown viruses are shown in Table 2. At least two independent TUGs was set as a minimum requirement to support each taxon. One exception to this was made for a single 2 649 nt long TUG of *B*. *terrestris*, contig_494, assembled from 2 905 reads that showed 42 percent identity to the RdRp of *Diaphorina citri*-associated C virus (DcACV). MEGAN analysis clustered this contig into *Tombusviridae* based on lower significant matches to viruses other than DcACV. Multiple sequence alignment (S3 Fig) to related tombusviruses and nodaviruses revealed the characteristic domains I-VIII of the (+) ssRNA viruses and the domain IV-VI consensus motif Dx_3_FDx_n_SGx_3_Tx_3_Nx_n_GDD [47]. Interestingly, this viral segment is related to the currently unclassified honey bee pathogen Chronic bee paralysis virus (CBPV).

**Table 2:** Undescribed viruses detected by RNA shotgun metagenomics sequencing of wild bees.

Virus group and host	Designation	Length	Top hit name^a^	Gene/ORF	ID^b^ (%)	QC^b^ (%)	E-value
Double-stranded DNA virus (*Nudiviridae*)							
*O*. *cornuta*	contig_19523	1157	KV	Gp119	49	80	9e-38
	contig_9159	1232	KV	Gp19-Like	38	61	6e-48
	contig_9125	915	OrNV	Orfd5	36	47	2e-17
	contig_11242	820	OrNV	Dnapol	49	92	5e-71
	389Z3:02184:02306	437	NpeNV	Gp003	43	96	4e-32
	389Z3:03390:02632	368	GbNV	Pif-2	60	92	2e-43
	30 others…						
Double-stranded RNA virus (*Totiviridae*)							
*O*. *cornuta*	contig_17891	382	LbTV	Coat protein	36	99	5e-08
	contig_4905	630	LbTV	RdRp	35	87	2e-25
	389Z3:00119:00163	325	LbTV	RdRp	49	52	1e-06
	389Z3:00301:01778	257	LbTV	Coat protein	42	89	9e-09
Unclassified (-) single-stranded RNA virus (*Bunyaviridae*)							
*O*. *cornuta*	contig_344	2519	WcCV-1	RdRp	45	86	5e-134
	contig_465	4272	WcCV-1	RdRp	42	85	0
	contig_8918	1109	WcCV-1	RdRp	46	20	9e-10
	389Z3:02074:00518	257	WhMV-1	RdRp	55	49	3e-07
*A*. *vaga*	contig_6424	333	XzMV	RdRp	28	98	2e-08
	contig_7678	627	XzMV	RdRp	40	63	1e-22
	contig_16490	297	RpBV	RdRp	57	89	2e-28
	389Z3:00261:00311	384	XzMV	RdRp	38	51	5e-06
Unclassified negative-sense, single-stranded RNA virus (Mononegavirales)							
*O*. *cornuta*	contig_2097	850	SyFV-1	Glycoprotein	29	46	2e-07
	contig_11562	1441	SgLV	RdRp	33	94	6e-63
	contig_10165	779	SgLV	RdRp	42	93	2e-36
	contig_8695	1077	SyFV-1	RdRp	53		1e-75
*A*. *vaga*	contig_10835	665	WhAV	RdRp	36	98	5e-15
	contig_12915	431	WhAV	RdRp	55	99	4e-33
	389Z3:03556:02011	287	WhAV	RdRp	52	99	2e-21
Positive-sense, single-stranded RNA virus (tombusnodaviruses)							
*B*. *terrestris*	contig_494	2649	DcACV	RdRp	42	39	8e-80
Unclassified positive-sense, single-stranded RNA virus (putative negeviruses)							
*B*. *terrestris*	contig_17335	441	Negev	RdRp	50		2e-06
	389Z3:00772:01069	305	Loreto	RdRp	38		9e-06
	389Z3:01341:02399	355	Negev	RdRp	38		1e-09
	389Z3:03397:01709	132	Piura	RdRp	64		7e-07
	389Z3:02407:00801	373	Loreto	RdRp	40		6e-12
*O*. *cornuta*	389Z3:00487:01970	247	Brejeira	Helicase	44		3e-08
	389Z3:00734:01113	406	Brejeira	RdRp	56		7e-25

^a^Abbreviations are: KV, Kallithea virus; OrNV, Oryctes rhinoceros nudivirus; NpeNV, Niliparvata lugens endogenous nudivirus; GbNV, Gryllus bimaculatus nudivirus; LbTV, Leptopilina boulardi totivirus; WcCV-1, Wuchang Cockroach Virus 1; XzMV, Xinzhou Mosquito Virus; RpBV, Rhinolophus pearsonii bunyavirus; SyFV-1, Shayang Fly Virus 1; SgLV, Shuangao Lacewing Virus; WhAV, Wuhan Ant Virus; DcACV, Diaphorina citri-associated C virus; RdRp, RNA-dependent RNA polymerase.

^b^Diamond blastx identity (ID) and query coverage (QC) percentages.

Thirty-six TUGs clustered in *Nudiviridae*, a family of insect-specific double-stranded DNA viruses. Two unassembled reads of *O*. *cornuta*, 389Z:02654:02477 and 389Z:03390:02632, matched the baculo_44 conserved domain pfam04631 of the *per os* infectivity factor 2 (pif-2). This protein is one of the four conserved proteins that are essential factors for baculoviruses to establish infections in insect hosts [48]. Contig_11242, length 820 nt, matched the DNA polymerase B protein and other TUGs matched various proteins of genome-sequenced exogenous nudiviruses such as *Oryctes rhinoceros* nudivirus, Kallithea virus, *Gryllus bimaculatus* nudivirus and *Niliparvata lugens* endogenous nudivirus (Table 2).

Four TUGs from *O*. *cornuta* had top hits to a dsRNA Toti-like virus from the parasitoid wasp *Leptopilina boulardi*. Seven TUGs, including 5 from *B*. *terrestris* and 2 from *O*. *cornuta*, matched to the proteins of negeviruses. Moreover, numerous TUGs matched proteins of (-) ssRNA viruses in *A*. *vaga*. Due to the low degree of evidence for these potential viruses, no reliable phylogenetic analysis could be made.

## Discussion

The eukaryote and viral diversity associated with four common wild bee species–*Bombus terrestris*, *Bombus pascuorum*, *Osmia cornuta* and *Andrena vaga—*was characterized in this study. We identified three of the known bumble bee parasites, including the trypanosome *C*. *bombi*, the neogregarine *A*. *bombi* and the nematode *S*. *bombi*. The trypanosome was identified in the *B*. *terrestris* and the *O*. *cornuta* metagenome, confirming the previous molecular detection of this parasite in *Osmia* [23]. The occurrence of *C*. *bombi* in *Osmia* suggests a broad host range. The trypanosome parasite can be vectored among insects in a pollinator community through shared flower visits [49]. The nematode *S*. *bombi* is a common parasite of queen bumble bees. The infective-stage fertilized female enters the host hemocoel during hibernation and everts its uterus to acquire nutrients directly from the hemolymph [50]. As the infection progresses, the host queen is sterilized by an as yet undescribed mechanism while tens of thousands of parasite progenies are raised. After dispersion from the host and subsequent mating, the impregnated female worm awaits the rare encounter of a new host and if successful, completes the parasite life cycle [51]. The high number of third-stage juvenile progenies that typically reside in the hemocoel explains the abundant detection of nematode-derived sequences in the *Bombus* metagenomes. Also, chances of catching an infected queen in the current sampling period (April-May) were relatively high since the ratio of infected to uninfected queens increases towards the end of spring and summer [51]. Notably, the *Sphaerularia* from this study was more related to a Japanese isolate than a UK isolate. Comparison of the 18S sequences deposited in genbank revealed that this UK isolate is more related to *Sphaerularia vespae*, a nematode isolated from the Japanese hornet *Vespa simillima* [52]. More sampling efforts in *Bombus* and other Hymenopteran hosts are necessary to obtain clarity on the taxonomic classification of current isolates as well as the host range of this remarkable endoparasite. Also, the molecular mechanism that underlies host sterility is worth future attention. The partial transcriptome data acquired here can facilitate this research. In addition, we detected a close relative to the cleptoparasitic mite *Chaetodactylus krombeini*, which is a major pest mite in the orchard pollinator *Osmia cornifrons* [53]. The mite hitchhikes on top of the bee abdomen to ultimately reach the rich pollen resources in the nest, on which it feeds. Bees are often overloaded with mites, impairing flight capacity and reproductive success. The storage mite *T*. *longior* putatively detected in this study has been reported from bumble bee nests but also many other habitats [54]. No endoparasitic or tracheal mites were found in *Bombus*. Either bumble bees did not carry tracheal mites or the parasites were removed by exclusion of the thoracic tagma which we intentionally left out in order to reduce the host transcriptome fraction.

A small subset of the wild bee metagenomes constituted viral entities associated with bees. We successfully characterized the representative genome sequence(s) for two viruses in *O*. *cornuta*, SRBV and GABV. Both are negative-sense ssRNA viruses and are distantly related to other arthropod viruses that were recently characterized [26]. In this study, we provide *in silico* evidence for a similar genomic organization to close relative viruses, terminal complementary regions in case of the GABV large segment and active replication of the virus by detecting reads of the replicative RNA strand although no solid evidence for replication in bee tissue was obtained. A recent transcriptome study in *O*. *cornuta* identified contigs originating from GABV in pooled tissue from bee antennae [28, 55]. Despite of the strong support for the ancestral origin of (-) ssRNA viruses in arthropods [28], the fact that this diverse group of viruses are also able to infect vertebrates and plants raises the question what are the primary hosts of SRBV and GABV. Whether SRBV, GABV and relative viruses are satisfied with insects as an exclusive host or whether the insect only acts as a temporary reservoir to reach the primary host (e.g. a plant species) remains open. Furthermore, we found additional sequences matching (-) ssRNA viral proteins in *A*. *vaga* but not in the social wild bee. Significant matches between phasmavirus nucleoproteins and sequence archives of eusocial *Bombus terrestris* and primitive eusocial *Exoneura robusta* are reported [28]. Together, these results support the presence of more (-) ssRNA viruses in bees that await characterization. Future steps including biological isolation and cell culture as well as *in vivo* infection studies should be considered to further unravel the physiological function of these viruses.

Three honey bee viruses BQCV, SBV and VDV-1 were detected albeit in very low concentration. The low abundance of reads likely reflects a low abundant presence in wild bees. Moreover, because all reads were of the genomic orientation, active replication of the honey bee viruses in wild bees could not be inferred from our data. We argue that BQCV, SBV and VDV-1 were merely associated with pollen and can circulate rapidly among pollinators by means of shared flower visits. This adds to the findings of Singh and co-authors who demonstrated the circulation of RNA viruses among bees through pollen-vectoring [22]. Another viral match included the RdRp of a tombusnodavirus related to the psyllid virus DcACV [56] and the honey bee CBPV. This was an unusual finding because these viruses lack a 3’ end polyA-tail [57] and are theoretically not retained by polyA+ mRNA enrichment. Either the RNA segment was present in very high copy numbers or the virus has a polyadenylated genome and hence differs significantly from its closest relatives. Attempts to identify a second RNA segment failed. Further, various viral sequences of *O*. *cornuta* supported the presence of a nudivirus. The family of *Nudiviridae* comprises arthropod-specific viruses that have a circular dsDNA genome ranging from 97K bp to 228K bp in size. The virus associated with *O*. *cornuta* showed highest homologies to the beetle virus *Oryctes rhinoceros nudivirus* which causes serious disease upon infection of the host gut and fat body tissues [58]. Last, fragmentary sequences matched proteins from a dsRNA virus and negeviruses. The latter is a recently proposed taxon of insect-specific viruses in mosquitoes and phlebotomine sand flies [59]. The significance of the fragmentary sequences is low and care should be taken when building further specifically on these findings. Nevertheless, it is possible that wild bees harbor a distinct set of insect-specific viruses that are to be revealed in the future. The strongest support, in our opinion, is presented here for SRBV and GABV, two new viruses in *O*. *cornuta* for which near-full length genome sequences were characterized.

## Supporting Information

S1 File Validation of the genomic sequence of SRBV. (SVG)

S2 File Validation of the L segment sequence of GABV. (SVG)

S3 File Validation of the M segment sequence of GABV. (SVG)

S4 File Validation of the S segment sequence of GABV. (SVG)

S5 File List of sequencing primers used for high-fidelity amplification and sanger sequencing. (XLSX)

S6 File Chimeric construct formation and homopolymers in Ion Torrent data. (DOCX)

S7 File RT-PCR screening of known bee pathogens. (DOCX)

S1 Fig Phylogenetic tree of SRBV and related viruses of the order Mononegavirales. (PDF)

S2 Fig Phylogenetic tree of GABV and related viruses of the family *Bunyaviridae* and *Arenaviridae*. (PDF)

S3 Fig Multiple sequence aligment of the RdRp conserved domains of a tombusnodavirus and related viruses. (PDF)
