# Supplementary material for: Unbiased RNA Shotgun Metagenomics in Social and Solitary Wild Bees Detects Associations with Eukaryote Parasites and New Viruses
Source: PLoS One. 2016 Dec 22;11(12):e0168456. doi: 10.1371/journal.pone.0168456 (PMC5179009; doi:10.1371/journal.pone.0168456)
Supplement: S6 File — (DOCX) [file pone.0168456.s006.docx]

Supplemental Information S6 File: polyA+ mRNA enrichment results and chimeric construct formation in Ion Torrent semiconductor sequencing

PolyA+ mRNA enrichment results

The ribosomal RNA load was reduced by enrichment of the polyA+ mRNA fraction of total RNA samples. To assess the effectiveness of this approach, RNA profiles before and after enrichment were compared by capillary electrophoresis (Figure S1). Enrichment clearly resulted in a strong reduction of rRNA peak sizes and a decrease in the rRNA/mRNA ratio. It should be mentioned that the low peak size of the LSU rRNA complies with the characteristic lability of the insect 28S rRNA subunit (see [63]). The 28S rRNA subunit bears an endogenous break that results in two equally sized fragments after RNA extraction upon extraction. The RNA integrity number (RIN) value which is a measure for RNA quality is therefore inapplicable insect total RNA.


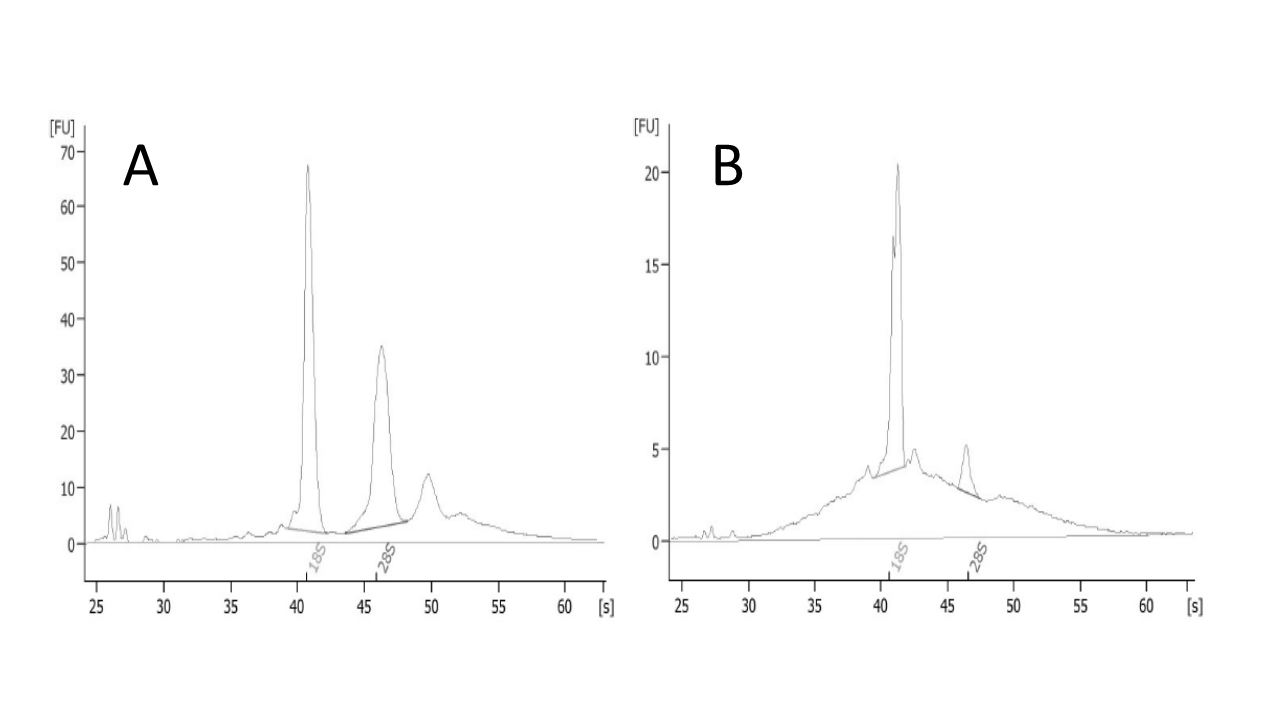


**Figure S1. Bioanalyzer electropherogram of total RNA (A) versus polyA+ mRNA enriched total RNA (B).** Results are only shown for *Bombus terrestris.* All samples had similar profiles except for the bigger size of the LSU peak in *Bombus* species compared to solitary bees. High peaks at 41 s and 46 s respectively represent the eukaryotic SSU and LSU ribosomal RNA species. In the polyA+ mRNA enriched total RNA sample, peak heights are strongly reduced. [FU] = signal intensity.

Chimeric construct formation in Ion Torrent semiconductor sequencing.

In multiple occurrences, a single Ion Torrent read matched two or more biologically unrelated transcripts. For example, read bter_389Z3:02844:01953 (452 bp) matched the 28S rRNA of *Sphaerularia bombi* (1..304) and the 60S ribosomal protein L7 of *Bombus terrestris* (303..452). Read bter_389Z3:02745:01544 (259 bp) matched the transcriptional regulator ATRX homolog of *B. terrestris* (1..133) and the UDP-glucoronic acid decarboxylase 2-like of the plant *Prunus mume* (131..259). Read ocor_389Z3:00920:02131 (428 bp) matched the tetratricopeptide repeat protein 7B of *Megachile rotundata* (4..190) and the 5.8S rRNA of the plant *Prunus* sp. (256..428). It was observed that the set of transcripts giving rise to chimeric reads lack sequence overlap in the region of the fusion boundary and that chimeric reads are unique. Both observations support the hypothesis that chimeric construct formation results from a random process rather than a sequence-directed hybridization and amplification process.

The second step of the Ion Torrent Whole Transcriptome Library protocol using the Ion Total RNA-Seq Kit is the ligation of adapters to the RNase III-fragmented RNA. Adapter ligation is a two-step procedure. First, a hybridization master mix containing Ion Adaptor Mix and Hybridization Solution is added to 50 ng of polyA+ RNA and fragments are allowed to hybridize to random “NNNNN” overhangs of the adapter molecules. The hybridization reaction is as follows: 10’ at 65°C followed by 5’ at 30°C. Second, a ligation master mix containing 2x Ligation Buffer and an undefined Ligation Enzyme Mix is added to the hybridization product. The ligation reaction is performed by 30’ incubation at 30°C.

Ion adapters should be added in excess molar concentrations compared to RNA inserts, allowing 1:1:1 hybridization and ligation of 5’ end adapter, RNA insert and 3’ end adapter, respectively. Flanking of the insert by two adapters is a prerequisite for it to be finally sequenced. If the adapter-insert ratio is suboptimal, a fraction of inserts will be either 5’-end adapted or 3’-end adapted instead of being flanked by both adapters. We argue that in the subsequent ligation step a 5’-end adapted insert is fused to a 3’-end adapted insert, giving rise to a randomly generated fusion insert flanked by both adapter species.

In multiple occurrences, chimeric constructs gave rise to misassembled chimeric contigs. It is evident that quantification of chimeric transcripts through metrics such as reads per kilo base per million mapped reads (RPKM) gives a distorted representation of the true transcript abundance in the sample and therefore quantification was not applicable in our study, unfortunately. Annotation of chimeric constructs by blast analysis favors the chimera part that receives the most significant match, i.e. the lowest evalue. In the case both parts of the chimera received comparable evalues, the chimera is allocated to a higher taxon by the lowest common ancestor principle in MEGAN. For example, read ocor_389Z3:00920:02131 (see above) was allocated to the taxon “Eukaryota” since the evalues for the ‘host’ part (6e-78) and ‘plant’ part (6e-76) were comparable. Because chimeric construct formation is considered random and the abundance of chimera was not overrepresented in our data sets, we still obtained a valid representation of the annotated metagenome. We tried to quantify chimera abundance and we also tried to filter out chimeras by adjusting MEGAN parameters, but this appeared a very difficult task with a high false positive rate.

Based on our experience with Ion Torrent sequencing, we can summarize that, despite of the flawless execution of the manufacturer’s protocol, the library preparation is sensitive to the introduction of technical errors that influence downstream bioinformatics analysis. We strongly advice to carry out an accurate estimation of the RNA concentration after fragmentation by capillary electrophoresis and to load fewer polyA+ RNA material than is recommended by the Ion Total RNA-Seq Kit V2 protocol.
