# Supplementary material for: Unbiased RNA Shotgun Metagenomics in Social and Solitary Wild Bees Detects Associations with Eukaryote Parasites and New Viruses
Source: PLoS One. 2016 Dec 22;11(12):e0168456. doi: 10.1371/journal.pone.0168456 (PMC5179009; doi:10.1371/journal.pone.0168456)
Supplement: S7 File — (DOCX) [file pone.0168456.s007.docx]

Supplemental Information S7 File: RT-PCR screening results of known honey bee pathogens

| **Sample** | **Viruses** | ***Crithidia* spp.** | **Neogregarinorida** | ***Nosema* sp.** | ***Ascosphaera* spp.** |
| --- | --- | --- | --- | --- | --- |
| *Bombus terrestris* | SBV | - | *Apicystis bombi* | *Nosema thomsoni* | - |
| *Bombus pascuorum* | - | - | - | - | - |
| *Osmia cornuta* | DWV | - | *Apicystis bombi* | *Nosema thomsoni* | *Ascosphaera sp.* |
| *Andrena vaga* | - | - | - | *Nosema thomsoni* | - |

|  | Primer name | Sequence (5'>3') | ref. |
| --- | --- | --- | --- |
| DWV | DWV-F1425 | CGTCGGCCTATCAAAG | [1] |
|  | DWV-B1806 | CTTTTCTAATTCAACTTCACC |  |
| BQCV | BQCV700-F | TGGTCAGCTCCCACTACCTTAAAC | [2] |
|  | BQCV700-R | GCAACAAGAAGAAACGTAAACCAC |  |
| LSV | LSVdeg-F | GCCWCGRYTGTTGGTYCCCCC | [3] |
|  | LSVdeg-R | GAGTGGCGGCGCSAGATAAAGT |  |
| AmFV | AmFV-BroN-F | CAGAGAATTCGGTTTTTGTGAGTG | [9] |
|  | AmFV-BroN-R | CATGGTGGCCAAGTCTTGCT |  |
| VdMLV | VdMLV-F | ATCCCTTTTCAGTTCGCT | [4] |
|  | VdMLV-R | AGAAGAGACTTCAAGGAC |  |
| SBV | SBV693F | GCACGTTTAATTGGGGATCA | [2] |
|  | SBV693R | CAGGTTGTCCCTTACCTCCA |  |
| *Apicystis* bombi | ApBF1 | CGTACTGCCCTGAATACTCCAG | [5] |
|  | ApBR1 | TGAAAGCGGCGTATACATGA |  |
| *Crithidia* bombi | 500Tr-CytB-F | TGTGGWGTKTGTTTAGC | [6] |
|  | 500Tr-CytB-R | CRTCWGAACTCATAAAATAATG |  |
| *Nosema* spp. | Nos-F | TATGCCGACGATGTGATATG | [7] |
|  | Nos-R | CACAGCATCCATTGAAAACG | [8] |
| *Ascosphaera* | KS25_513f | TGGACCTTTCCTTCTGGGGA | this study |
|  | KS26_1194r | CAAACGTCGACCGGGCTATT | this study |

REFERENCE LIST

1. Forsgren E, de Miranda JR, Isaksson M, Wei S, Fries I. Deformed wing virus associated with Tropilaelaps mercedesae infesting European honey bees (Apis mellifera). Exp Appl Acarol. 2009;47(2):87-97. doi: 10.1007/s10493-008-9204-4. PubMed PMID: 18941909.

2. Singh R, Levitt AL, Rajotte EG, Holmes EC, Ostiguy N, Vanengelsdorp D, et al. RNA Viruses in Hymenopteran Pollinators: Evidence of Inter-Taxa Virus Transmission via Pollen and Potential Impact on Non-Apis Hymenopteran Species. Plos One. 2010;5(12). doi: ARTN e14357

10.1371/journal.pone.0014357. PubMed PMID: WOS:000285578000004.

3. Ravoet J, Maharramov J, Meeus I, De Smet L, Wenseleers T, Smagghe G, et al. Comprehensive bee pathogen screening in Belgium reveals Crithidia mellificae as a new contributory factor to winter mortality. PLoS One. 2013;8(8):e72443. doi: 10.1371/journal.pone.0072443. PubMed PMID: 23991113; PubMed Central PMCID: PMCPMC3753275.

4. Gauthier L, Ravallec M, Tournaire M, Cousserans F, Bergoin M, Dainat B, et al. Viruses associated with ovarian degeneration in Apis mellifera L. queens. PLoS One. 2011;6(1):e16217. doi: 10.1371/journal.pone.0016217. PubMed PMID: 21283547; PubMed Central PMCID: PMCPMC3026828.

5. Meeus I, de Graaf DC, Jans K, Smagghe G. Multiplex PCR detection of slowly-evolving trypanosomatids and neogregarines in bumblebees using broad-range primers. J Appl Microbiol. 2010;109(1):107-15. doi: 10.1111/j.1365-2672.2009.04635.x. PubMed PMID: 20015206.

6. Ravoet J, De Smet L, Meeus I, Smagghe G, Wenseleers T, de Graaf DC. Widespread occurrence of honey bee pathogens in solitary bees. J Invertebr Pathol. 2014;122:55-8. doi: 10.1016/j.jip.2014.08.007. PubMed PMID: 25196470.

7. Fernandez JM, Puerta F, Cousinou M, Dios-Palomares R, Campano F, Redondo L. Asymptomatic presence of Nosema spp. in Spanish commercial apiaries. J Invertebr Pathol. 2012;111(2):106-10. doi: 10.1016/j.jip.2012.06.008. PubMed PMID: 22820066.

8. Higes M, Martin R, Meana A. Nosema ceranae, a new microsporidian parasite in honeybees in Europe. J Invertebr Pathol. 2006;92(2):93-5. doi: 10.1016/j.jip.2006.02.005. PubMed PMID: 16574143.

9. Hartmann, U., Gauthier, L., Cornman, R.S., Chen, Y.P., Evans, J.D., Charriere, J.D., Forsgren, E., de Miranda, J.R., Neumann, P. Partial sequence and prevalence of Apis mellifera filamentous virus (AmFV), a honeybee DNA virus related to ascoviruses and baculoviruses. 2012. Ref Type: Unpublished Work
