# Supplementary material for: Unbiased RNA Shotgun Metagenomics in Social and Solitary Wild Bees Detects Associations with Eukaryote Parasites and New Viruses
Source: PLoS One. 2016 Dec 22;11(12):e0168456. doi: 10.1371/journal.pone.0168456 (PMC5179009; doi:10.1371/journal.pone.0168456)
Supplement: S1 Fig — (PDF) [file pone.0168456.s008.pdf]

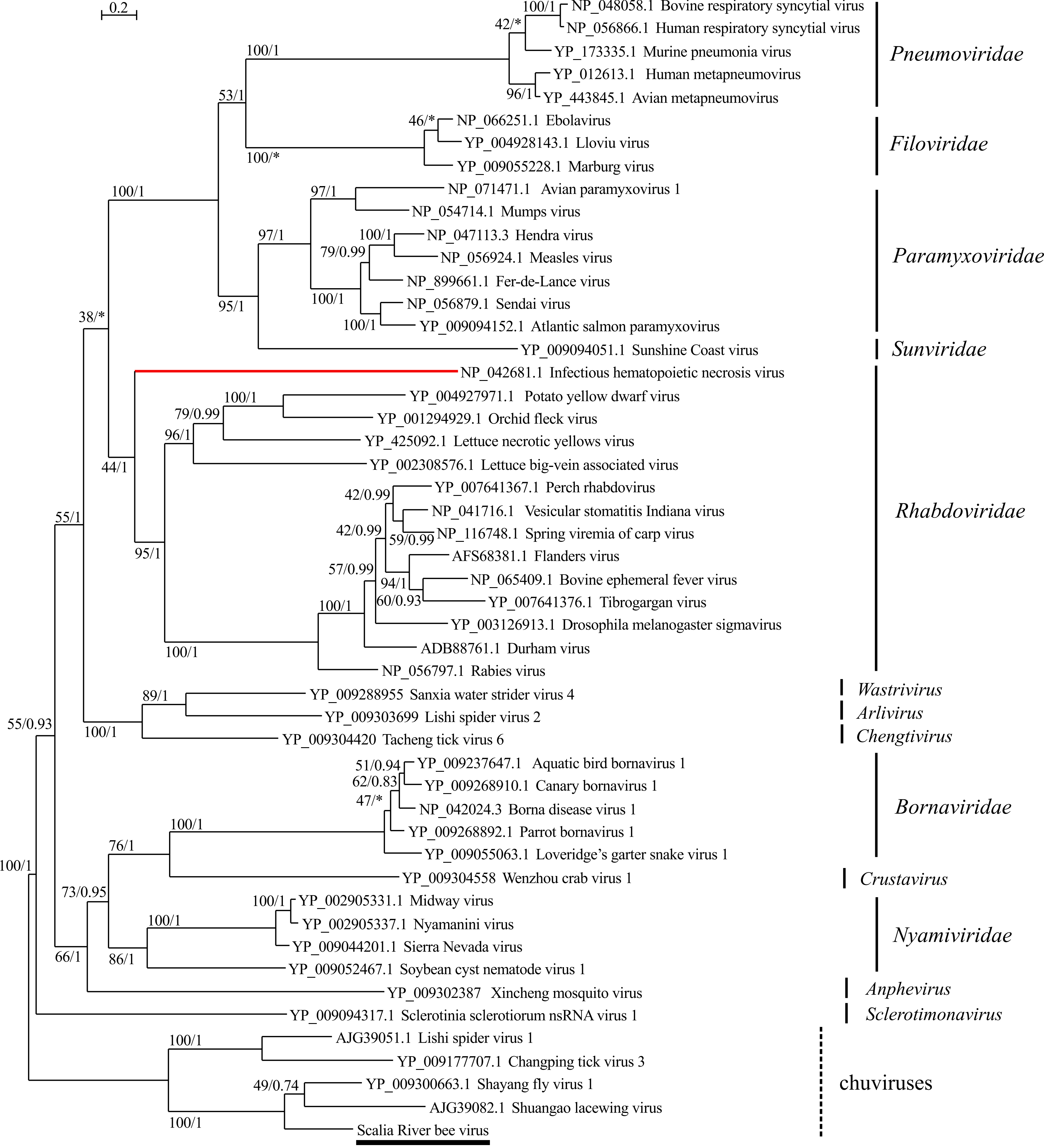

S1 Fig. Phylogenetic tree of Scalia River bee virus (SRBV) and related viruses of the order Mononegavirales. The viruses included in this tree correspond to the type species of each genus according to the latest taxonomic classification by the ICTV. Multiple sequence aligment of the entire RdRp protein was done with MAFFT and the E-INS-i algorithm which is optimal for aligning multiple conserved motifs embedded in long unalignable regions. Non-informative positions (t-coffee TCS columns score <5) were removed. The final aligment contained 545 positions. The optimal substitution model out of 120 models was selected using ProtTest3. Maximum Likelihood (PhyML in SeaView) and Bayesian Inference (MrBayes) analyses were performed under the LG+I+G+F model. For BI, two parallel mcmcmc runs were run beyond convergence (split freq < 0.01) for 1,000,000 generations. The consensus tree was summarized without 10% rel burn-in. The base tree presented here is the consensus topology of Maximum Likelihood analysis (1000 bootstrap replicates). Branches are labeled with ML % values/bayesian posterior probabilities. In case of conflict topology, only the ML % value is given. The branch separating chuviruses from the other Mononegavirales was selected as root. Bayesian analysis classified the novirhabdovirus branch (red) outside the clade of Rhabdoviridae and together with Paramyxoviridae (not illustrated in the ML tree). SRBV clustered among other chuviruses, more related to ShFV-1 and SgLV.
