# Supplementary material for: Unbiased RNA Shotgun Metagenomics in Social and Solitary Wild Bees Detects Associations with Eukaryote Parasites and New Viruses
Source: PLoS One. 2016 Dec 22;11(12):e0168456. doi: 10.1371/journal.pone.0168456 (PMC5179009; doi:10.1371/journal.pone.0168456)
Supplement: S2 Fig — (PDF) [file pone.0168456.s009.pdf]

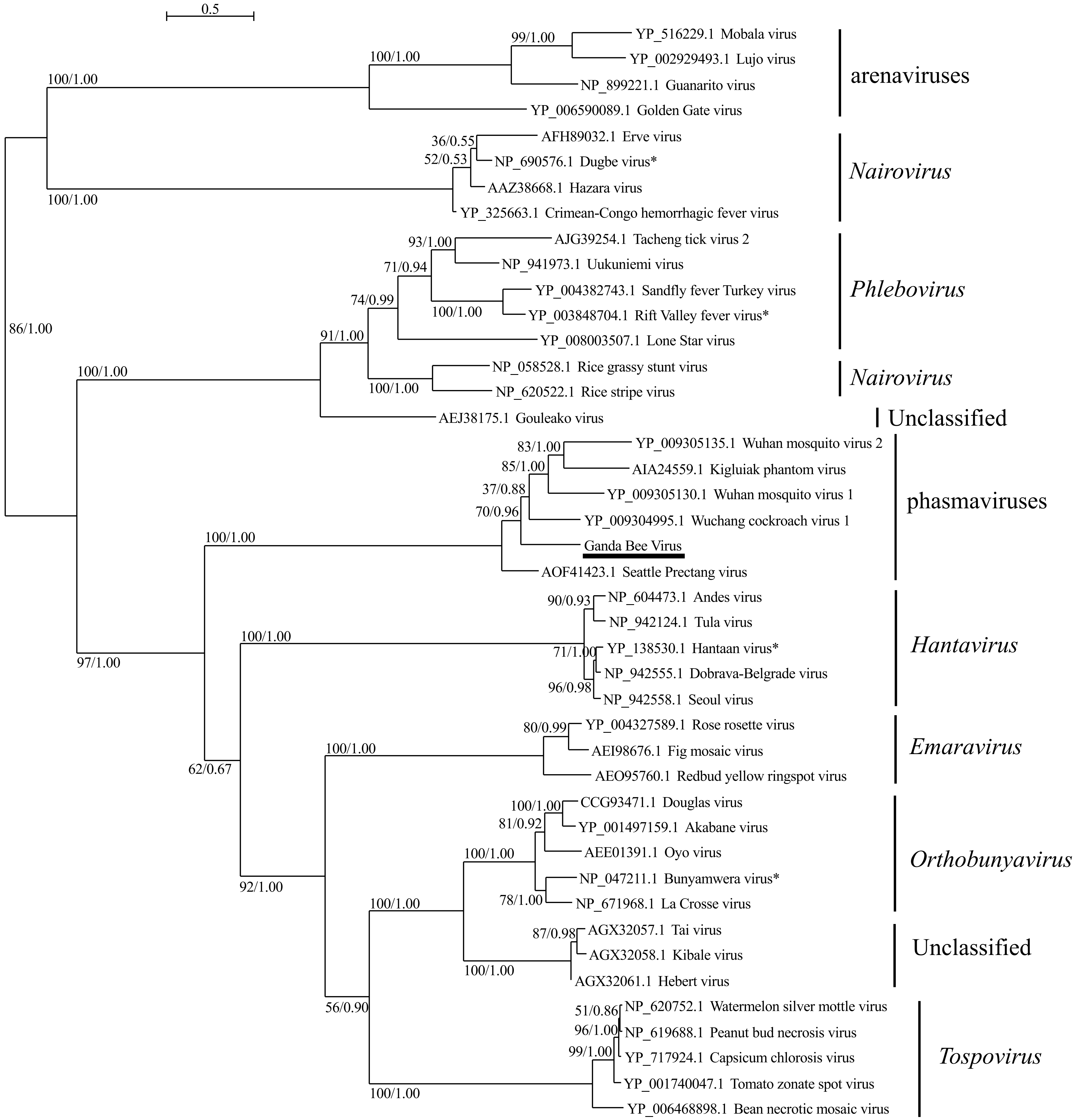

S2 Fig. Phylogenetic tree of Ganda bee virus (GABV) and related viruses of the families Bunyaviridae and Arenaviridae. Viruses representing the type species of a genus according to the latest taxonomic classification by the ICTV are marked with an asterisk. Multiple sequence aligment of the full RdRp protein was done with MAFFT and the E-INS-i algorithm which is optimal for aligning multiple conserved motifs embedded in long unalignable regions. Non-informative positions (t-coffee TCS columns score <5) were removed. The final aligment contained 512 positions. The optimal substitution model out of 120 models was selected using ProtTest3. Maximum Likelihood (PhyML in SeaView) and Bayesian Inference (MrBayes) analyses were performed under the LG+I+G+F model. For BI, two parallel mcmcmc runs were run beyond convergence (split freq < 0.01) for 1,000,000 generations. The consensus tree was summarized without 10% rel burn-in. The base tree presented here is the consensus topology of Maximum Likelihood analysis (1000 bootstrap replicates). Branches are labeled with ML % values/bayesian posterior probabilities. In case of conflict topology, only the ML % value is given. The branch separating arenaviruses and the genus Nairovirus was selected as root. GABV was clustered among the informal group phasmaviruses.
