# Supplementary material for: Unbiased RNA Shotgun Metagenomics in Social and Solitary Wild Bees Detects Associations with Eukaryote Parasites and New Viruses
Source: PLoS One. 2016 Dec 22;11(12):e0168456. doi: 10.1371/journal.pone.0168456 (PMC5179009; doi:10.1371/journal.pone.0168456)
Supplement: S3 Fig — (PDF) [file pone.0168456.s010.pdf]

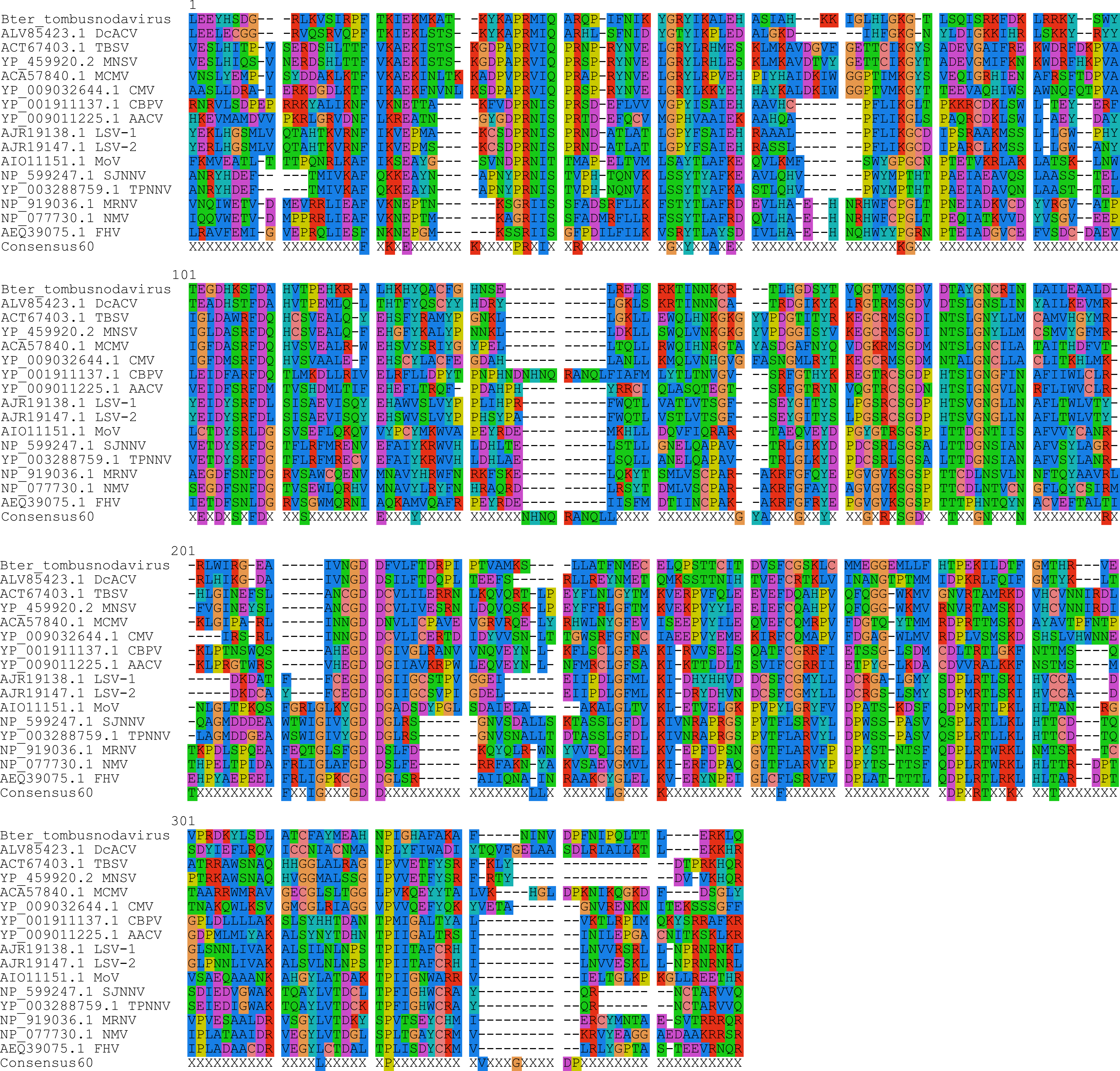
1
Bter_tombusnodavirus LEEYHSDG-- -RLKVSIRPF TKIEKMKAT- -KYKAPRMIQ ARQP-IFNIK YGRYIKALEH ASIAH---KK IGLHLGKG-T LSQISRKFDK LRRKY--SWY
ALV85423.1 DcACV LEELECGG-- -RVQSRVQPF TKIEKLSTS- -KYKAPRMIQ ARHL-SFNID YGTYIKPLED ALGKD----- --IHFGKG-N YLDIGKKIHR LSKKY--RYY
ACT67403.1 TBSV VESLHITP-V SERDSHLTTF VKAEKISTS- KGDPAPRVIQ PRNP-RYNVE LGRYLRHMES KLMKAVDGVF GETTCIKGYS ADEVGAIFRE KWDRFDKPVA
YP_459920.2 MNSV VESLHIQS-V NERDSHLTTF VKAEKISTS- KGDPAPRVIQ PRSP-RYNVE LGRYLRHLES KLMKAVDTVY GETTCIKGYT ADEVGMIFKN KWDRFHKPVA
ACA57840.1 MCMV VNSLYEMP-V SYDDAKLKTF VKAEKINLTK KADPVPRVIQ PRAP-RYNVE LGRYLRPVEH PIYHAIDKIW GGPTIMKGYS VEQIGRHIEN AFRSFTDPVA
YP_009032644.1 CMV AASLLDRA-I ERKDGDLKTF IKAEKFNVNL KSDPAPRVIQ PRSP-RYNVE LGRYLKKYEH HAYKALDKIW GGPTVMKGYT TEEVAQHIWS AWNQFQTPVA
YP_001911137.1 CBPV RNRVLSDPEP RRKYALIKNF VKNETTA--- -KFVDPRNIS PRSD-EFLVV VGPYISAIEH AAVHC----- --PFLIKGLT PKKRCDKLSW L-TEY--ERF
YP_009011225.1 AACV HKEVMAMDVV PKRLGRVDNF LKREATN--- -GYGDPRNIS PRTD-EFQCV MGPHVAAIEK AAHQA----- --PFLIKGLS PAQRCDKLSW L-AEY--DAY
AJR19138.1 LSV-1 YEKLHGSMLV QTAHTKVRNF IKVEPMA--- -KCSDPRNIS PRND-ATLAT LGPYFSAIEH RAAAL----- --PFLIKGCD IPSRAAKMSS L-LGW--PHY
AJR19147.1 LSV-2 YERLHGSMLV QTAHTKVRNF IKVEPMS--- -KCSDPRNIS PRND-ATLAT LGPYFSAIEH RASSL----- --PFLIKGCD IPARCLKMSS L-LGW--ANY
AIO11151.1 MoV FKMVEATL-T TTPQNRLKAF IKSEAYG--- -SVNDPRNIT TMAP-ELTVM LSAYTLAFKE QVLKMF---- --SWYGPGCN PTETVKRLAK LATSK--LNW
NP_599247.1 SJNNV ANRYHDEF-- ---TMIVKAF QKKEAYN--- -APNYPRNIS TVPH-TQNVK LSSYTYAFKE AVLQHV---- --PWYMPTHT PAEIAEAVQS LAASS--TEL
YP_003288759.1 TPNNV ANRYHDEF-- ---TMIVKAF QKKEAYN--- -APNYPRNIS TVPH-NQNVK LSSYTYAFKA STLQHV---- --PWYMPTHT PAEIADAVQN LAAST--TEL
NP_919036.1 MRNV VNQIWETV-D MEVRRLIEAF VKNEPTN--- ---KSGRIIS SFADSRFLLK FSTYTLAFRD EVLHA-E--H NRHWFCPGLT PNEIADKVCD YVRGV--ATP
NP_077730.1 NMV IQQVWETV-D MPPRRLIEAF VKNEPTM--- ---KAGRIIS SFADMRFLLR FSSYTLAFRD QVLHA-E--H NRHWFCPGLT PEQIATKVVD YVSGV--EEP
AEQ39075.1 FHV LRAVFEMI-G VEPRQLIESF NKNEPGM--- ---KSSRIIS GFPDILFILK VSRYTLAYSD IVLHA-E--H NQHWYYPGRN PTEIADGVCE FVSDC-DAEV
Consensus60 XXXXXXXXXX XXXXXXXXXF XKXEXXXXXX KXXXXPRXIX XRXXXXXXXX XGXYXXAXEX XXXXXXXXXX XXXXXXKGXX XXXXXXXXXX XXXXXXXXXX
101
Bter_tombusnodavirus TEGDHKSFDA HVTPEHKR-A LHKHYQACFG HNSE------ -----LRELS RKTINNKCR- --TLHGDSYT VQGTVMSGDV DTAYGNCRIN LAILEAALD-
ALV85423.1 DcACV TEADHSTFDA HVTPEMLQ-L THTFYQSCYY HDRY------ -----LGKLS KRTINNNCA- --TRDGIKYK IRGTRMSGDV DTSLGNSLIN YAILKEVMR-
ACT67403.1 TBSV IGLDAWRFDQ HCSVEALQ-Y EHSFYRAMYP GNKL------ -----LGKLL EWQLHNKGKG YVPDGTITYR KEGCRMSGDI NTSLGNYLLM CAMVHGYMR-
YP_459920.2 MNSV IGLDASRFDQ HCSVEALQ-F EHGFYKALYP NNKL------ -----LDKLL SWQLVNKGKG YVPDGGISYV KEGCRMSGDI NTSLGNYLLM CSMVYGFMR-
ACA57840.1 MCMV IGFDASRFDQ HVSVEALR-W EHSVYSRIYG YPEL------ -----LTQLL RWQIHNRGTA YASDGAFNYQ VDGKRMSGDM NTSLGNCILA TAITHDFVT-
YP_009032644.1 CMV IGFDMSRFDQ HVSVAALE-F EHSCYLACFE GDAH------ -----LANLL KMQLVNHGVG FASNGMLRYT KEGCRMSGDM NTALGNCLLA CLITKHLMK-
YP_001911137.1 CBPV LEIDFARFDQ TLMKDLLRIV ELRFLLDPYT PNPHNDNHNQ RANQLFIAFM LYTLTNVGV- --SRFGTHYK REGTRCSGDP HTSIGNGFIN AFIIWLCLR-
YP_009011225.1 AACV VEIDFSRFDM TVSHDMLTIF EHEFLTRQF- PDAHPH---- -----YRRCI QLASQTEGT- --SKFGTRYN VQGTRCSGDN HTSIGNGVLN RFLIWVCLR-
AJR19138.1 LSV-1 YEIDYSRFDL SISAEVISQY EHAWVSLVYP PLIHPR---- -----FWQTL VATLVTSGF- --SEYGITYS LPGSRCSGDP HTSIGNGLLN AFLTWLVTY-
AJR19147.1 LSV-2 YEIDYSRFDL SISAEVISQY EHSWVSLVYP PHSYPA---- -----FWQTL VSTLVTSGF- --SEYGITYS LPGSRCSGDP HTSVGNGLLN AFLTWLVTY-
AIO11151.1 MoV LCTDYSRLDG SVSEFLQKQV VYPCYMKWVA PEYRDE---- -----MKHLL DQVFIQRAR- --TAEQVEYD PGYGTRSGSP ITTDGNTIIS AFVVYCANR-
NP_599247.1 SJNNV VETDYSKFDG TFLRFMRENV EFAIYKRWVH LDHLTE---- -----LSTLL GNELQAPAV- --TRLGIKYD PDCSRLSGSA LTTDGNSIAN AFVSYLAGR-
YP_003288759.1 TPNNV VETDYSKFDG TFLRFMRECV EFAIYKRWVH LDHLAE---- -----LSQLL ANELQAPAV- --TRLGLKYD PDCSRLSGSA LTTDGNSIAN AFVSYLANR-
NP_919036.1 MRNV AEGDFSNFDG RVSAWCQENV MNAVYHRWFN RKFSKE---- -----LQKYT SMLVSCPAR- -AKRFGFQYE PGVGVKSGSP TTCDLNSVLN NFTQYAAVRL
NP_077730.1 NMV SEGDFSNFDG TVSEWLQRHV MNAVYLRYFN HRAQRD---- -----LRSYT DMLVSCPAR- -AKRFGFAYD AGVGVKSGSP TTCDLNTVCN GFLQYCSIRM
AEQ39075.1 FHV IETDFSNLDG RVSGWMQRNI AQKAMVQAFR PEYRDE---- -----IISFM DTIINCPAK- -AKRFGFRYE PGVGVKSGSP TTTPHNTQYN ACVEFTALTI
Consensus60 XEXDXSXFDX XXSXXXXXXX EXXXYXXXXX XXXXXXNHNQ RANQLLXXXX XXXXXXXXXG YAXXXGXXYX XXGXRXSGDX XTXXGNXXXN XXXXXXXXRX
201
Bter_tombusnodavirus -RLWIRG-EA -----IVNGD DFVLFTDRPI PTVAMKS--- -LLATFNMEC ELQPSTTCIT DVSFCGSKLC MMEGGEMLLF HTPEKILDTF GMTHR---VE
ALV85423.1 DcACV -RLHIKG-DA -----IVNGD DSILFTDQPL TEEFS----- RLLREYNMET QMKSSTTNIH TVEFCRTKLV INANGTPTMM IDPKRLFQIF GMTYK---LT
ACT67403.1 TBSV -HLGINEFSL -----ANCGD DCVLILERRN LKQVQRT-LP EYFLNLGYTM KVERPVFQLE EVEFDQAHPV QFQGG-WKMV GNVRTAMRKD VHCVNNIRDL
YP_459920.2 MNSV -FVGINEYSL -----ANCGD DCVLIVESRN LDQVQSK-LP EYFFRLGFTM KVEKPVYYLE EIEFCQAHPV QFQGG-WKMV RNVRTAMSKD VHCVNNIRDI
ACA57840.1 MCMV -KLGIPA-RL -----INNGD DNVLICPAVE VGRVRQE-LY RHWLNYGFEV ISEEPVYILE QVEFCQMRPV FDGTQ-YTMM RDPRTTMSKD AYAVTPFNTP
YP_009032644.1 CMV ----IRS-RL -----INNGD DCVLICERTD IDYVVSN-LT TGWSRFGFNC IAEEPVYEME KIRFCQMAPV FDGAG-WLMV RDPLVSMSKD SHSLVHWNNE
YP_001911137.1 CBPV -KLPTNSWQS -----AHEGD DGIVGLRANV VNQVEYN-L- KFLSCLGFRA KI-RVVSELS QATFCGRRFI ETSSG-LSDM CDLTRTLGKF NTTMS----Q
YP_009011225.1 AACV -KLPRGTWRS -----VHEGD DGIIAVKRPW LEQVEYN-L- NFMRCLGFSA KI-KTTLDLT SVIFCGRRII ETPYG-LKDA CDVVRALKKF NSTMS----M
AJR19138.1 LSV-1 -----DKDAT F----FCEGD DGIIGCSTPV GGEI------ EIIPDLGFML KI-DHYHHVD DCSFCGMYLL DCRGA-LGMY SDPLRTLSKI HVCCA----D
AJR19147.1 LSV-2 -----DKDCA Y----FCEGD DGIIGCSVPI GDEL------ EIIPDLGFML KI-DRYDHVN DCSFCGMYLL DCRGS-LSMY SDPMRTLSKI HVCCA----D
AIO11151.1 MoV -NLGLTPKQS FGRLGLKYGD DGADSDYPGL SDAIELA--- --AKALGLTV KL-ETVELGK PVPYLGRYFV DPATS-KDSF QDPMRTLPKL HLTAN---RG
NP_599247.1 SJNNV -QAGMDDDEA WTWIGIVYGD DGLRS----- -GNVSDALLS KTASSLGFDL KIVNRAPRGS PVTFLSRVYL DPWSS-PASV QSPLRTLLKL HTTCD---TQ
YP_003288759.1 TPNNV -LAGMDDGEA WSWIGIVYGD DGLRS----- -GNVSNALLT DTASSLGFDL KIVNRAPRGS PVTFLARVYL DPWSS-PASV QSPLRTLLKL HTTCD---TQ
NP_919036.1 MRNV TKPDLSPQEA FEQTGLSFGD DSLFD----- -KQYQLR-WN YVVEQLGMEL KV-EPFDPSN GVTFLARVFP DPYST-NTSF QDPLRTWRKL NMTSR---TC
NP_077730.1 NMV THPELTPIDA FRLIGLAFGD DSLFE----- -RRFAKN-YA KVSAEVGMVL KI-ERFDPAQ GITFLARVYP DPYTS-TTSF QDPLRTWRKL HLTTR--DPT
AEQ39075.1 FHV EHPYAEPEEL FRLIGPKCGD DGLSR----- -AIIQNA-IN RAAKCYGLEL KV-ERYNPEI GLCFLSRVFV DPLAT-TTTL QDPLRTLRKL HLTAR--DPT
Consensus60 TXXXXXXXXX FXXIGXXXGD DXXXXXXXXX XXXXXXXLLX XXXXXLGXXX KXXXXXXXXX XXXFXXXXXX XXXXXXXXXX XDPXRTXXKX XXTXXXXXXX
301
Bter_tombusnodavirus VPRDKYLSDL ATCFAYMEAH NPIGHAFAKA F-----NINV DPFNIPQLTT L----ERKLQ
ALV85423.1 DcACV SDYIEFLRQV ICCNIACNMA NPLYFIWADI YTQVFGELAA SDLRIAILKT L----EKKHR
ACT67403.1 TBSV ATRRAWSNAQ HHGGLALRAG IPVVETFYSR F-KLY----- ---------- --DTPRKHQR
YP_459920.2 MNSV PTRKAWSNAQ HVGGMALSSG IPVVETFYSR F-RTY----- ---------- --DV-VKHQR
ACA57840.1 MCMV TAARRWMRAV GECGLSLTGG LPVKQEYYTA LVK----HGL DPKNIKQGKD F----DSGLY
YP_009032644.1 CMV TNAKQWLKSV GMCGLRIAGG VPVVQEFYQK YVETA----- --GNVRENKN ITEKSSSGFF
YP_001911137.1 CBPV GPLDLLLLAK SLSYHHTDAN TPMIGALTYA L--------- --VKTLRPIM QKYSRRAFKR
YP_009011225.1 AACV GDPMLMLYAK ALSYNYTDHN TPIIGALTRS I--------- --INILEPGA CNITKSKLKR
AJR19138.1 LSV-1 GLSNNLIVAK ALSILNLNPS TPIITAFCRH I--------- --LNVVRSRL L-NPRNRNKL
AJR19147.1 LSV-2 GLPNNLIVAK ALSVLNLNPS TPIITAFCRH I--------- --LNVVESKL L-NPRNRNRL
AIO11151.1 MoV VSAEQAAANK AHGYLATDAK TPIIGNWARR V--------- --IELTGLKP KGLLREETHR
NP_599247.1 SJNNV SDIEDVGWAK TQAYLVTDCL TPFIGHWCRA Y--------- --QR------ --NCTARVVQ
YP_003288759.1 TPNNV SEIEDIGWAK TQAYLVTDCK TPFIGHWCRA Y--------- --QR------ --NCTARVVQ
NP_919036.1 MRNV VPVESAALDR VSGYLVTDKY SPVTSEYCHM I--------- --ERCYMNTA E-SVTRRRQR
NP_077730.1 NMV IPLATAAIDR VEGYLVTDGL SPLTGAYCRM V--------- --KRVYEAGG AEDAAKRRSR
AEQ39075.1 FHV IPLADAACDR VEGYLCTDAL TPLISDYCKM V--------- --LRLYGPTA S-TEEVRNQR
Consensus60 XXXXXXXXXX XXXXLXXXXX XPXXXXXXXX XVXXXGXXXX DPXXXXXXXX XXXXXXXXXX
